# CT041 CAR T cell therapy for Claudin18.2-positive metastatic pancreatic cancer

**DOI:** 10.1186/s13045-023-01491-9

**Published:** 2023-09-09

**Authors:** Changsong Qi, Tong Xie, Jun Zhou, Xicheng Wang, Jifang Gong, Xiaotian Zhang, Jian Li, Jiajia Yuan, Chang Liu, Lin Shen

**Affiliations:** 1 Department of Early Drug Development Center, Key Laboratory of Carcinogenesis and Translational Research (Ministry of Education), Peking University Cancer Hospital & Institute, Beijing, China; 2 Department of Gastrointestinal Oncology, Key Laboratory of Carcinogenesis and Translational Research (Ministry of Education), Peking University Cancer Hospital & Institute, Beijing, China

## Abstract

Pancreatic cancer lacks effective therapy. Here, we reported two metastatic pancreatic cancer patients administrated with Claudin 18.2 (CLDN 18.2) CART therapy after the failure of standard therapy (NCT04581473 and NCT03874897). In case 1, with CLDN 18.2 expression of 2+, 70%, 250 × 10^6^ cells were infused after lymphodepletion. Grade 1 cytokine release syndrome (CRS) occurred on d1 which was later controlled by tocilizumab. Partial response (PR) was achieved according to RECIST v1.1, with great shrinkage of lung metastasis. An increasing CD8+ T cell and Treg cells and declining CD4+ T cell and B cell were observed. In case 2, IHC result of ClDN18.2 showed 3+, 60%. 250 × 10^6^ CLDN18.2 CART cells were subsequently administered. Patient experienced grade 2 CRS, which was controlled with tocilizumab. Target lesions of lung metastasis further achieved complete response. Similar increasing CD8+ T cell and Treg cell was detected from peripheral blood. Elevating IL-8 and declining TGF-β1 were also observed. The tumor is still under well control until the last follow-up on July 18, 2023.

**Supplementary Information:**

The online version contains supplementary material available at 10.1186/s13045-023-01491-9.

**To the editor**:

Pancreatic cancer lacks effective treatment, and the situation gets worse along the treatment line advances. CAR-T therapy is promising in treating hematologic malignancies; however, the efficacy in solid tumor is still limited according to previously reported clinical trials [1].

Claudin18.2 (CLDN18.2), an isoform of the tight junction protein CLDN18, is highly expressed in pancreatic cancer [2]. We found favorable response rate of CT041, a genetically engineered autologous T cells expressing CLDN18.2-targeted CAR, in previously treated digestive system malignances [3]. Here, we present two metastatic pancreatic cancer patients who received CT041 infusion, along with their changes in peripheral blood biomarkers.

## Case 1

After biopsy and PET-CT scan, a 58-year-old woman was diagnosed with pancreatic cancer with lung and lymph nodes metastasis. First-line nab-paclitaxel plus gemcitabine and second-line irinotecan-liposome plus 5-flurouracil were failed before she was enrolled in CT041 phase 1 clinical trial (Additional file 4: Figure S4), after confirming 2+, 70% expressing of CLDN18.2 (Additional file 1: Figure S1).

**Fig. 1:**
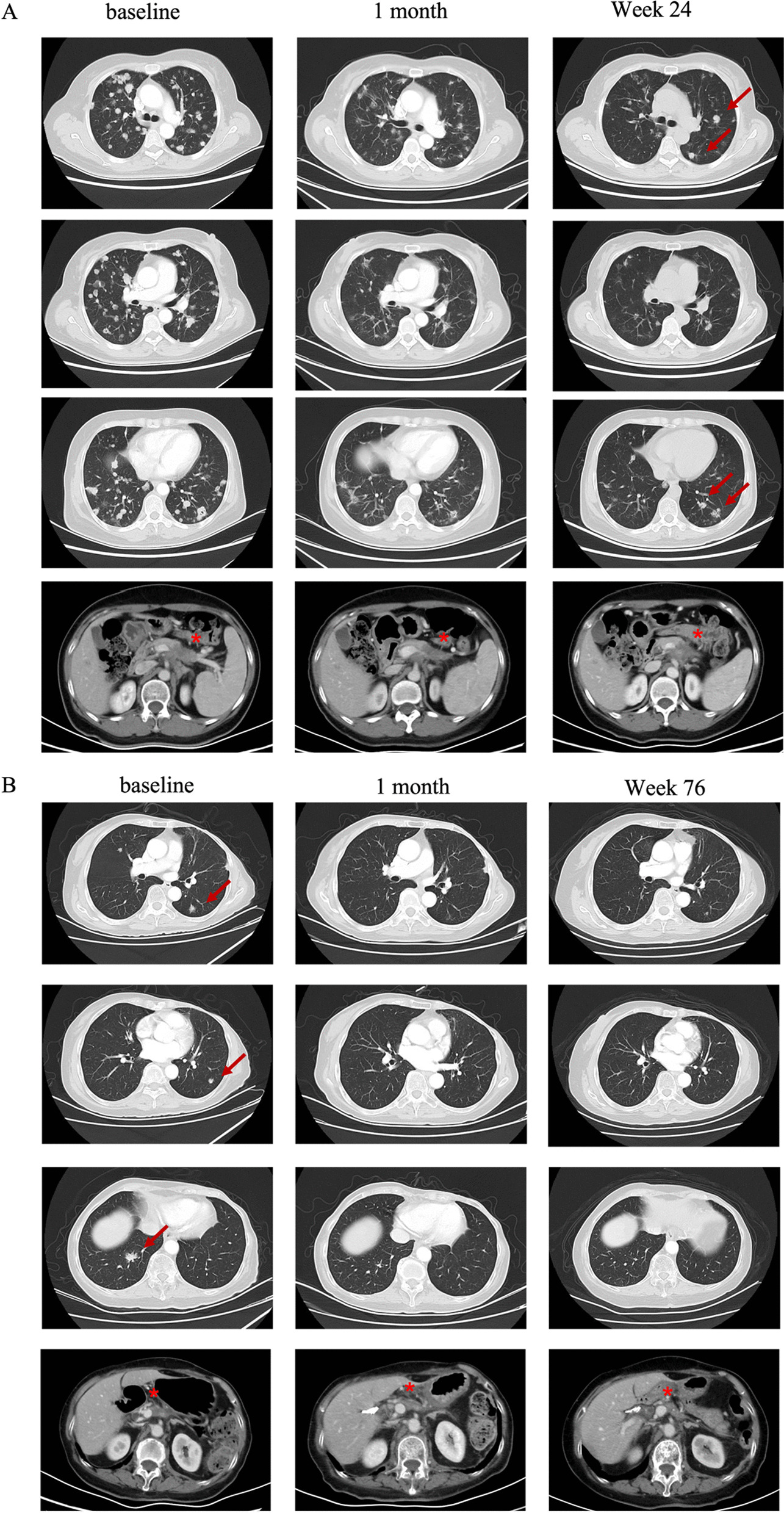
Radiological evaluation of lung lesions in case 1 (**A**) and case 2 (**B**). Red arrow in **A** showed the progressed lesion; red arrow in **B** indicated the target lesion. * indicated the primary lesion

One cycle of FOLFIRI was bridged on August 26, 2021. After lymphodepletion consisting of fludarabine, cyclophosphamide, and nab‐paclitaxel, a CT041 dose of 250 × 10^6^ cells was infused on September 22 (Additional file 2: Figure S2; Additional file 6: methods). Grade 1 cytokine release syndrome (CRS) occurred on D1 and upgraded to grade 2 on D3, until the administration of tocilizumab (Additional file 3: Figure S3). Partial response (PR) was achieved according to RECIST v1.1 (Fig. 1A), and liver lesion progressed on March 8, 2022, and the patient died due to disease progression on July 23, 2022.

**Fig. 2:**
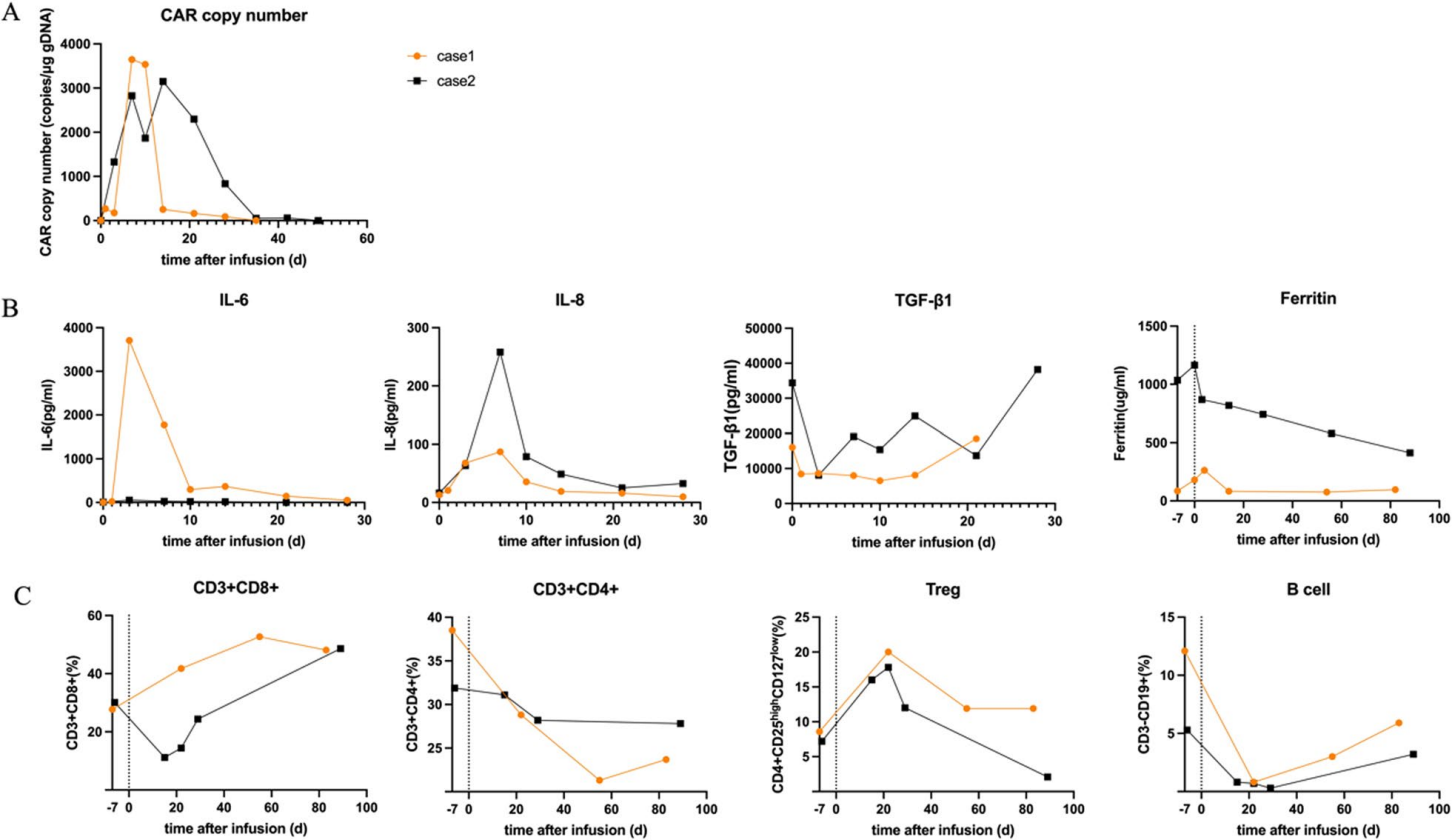
Dynamic changes of **A** CLDN18.2 CAR copy numbers, **B** cytokine levels and **C** peripheral blood lymphocyte subsets according to the time after CT041 infusion

The peripheral blood CLDN18.2 CAR copy reached peak on D7 and dropped below the limit of quantification after week 4. Fluorescence-activated cell sorting (FACS) showed a decrease in CD4+ T cells and B cells and an increase in CD8+ T cells and Treg cells. CD8+ T cells occupied nearly half of the total lymphocytes in the next 3 months. In contrast, CD4+ T cells remained at lower levels, but gradually increased for 3 months after CT041 infusion (Additional file 5: Table S1). Cytokine analysis showed a significant increase of IL-6 level since D3 and a significant reduction by week 4, contradictory to TGF-β1 and Treg (Fig. 2).

## Case 2

A 75-year-old woman underwent surgery due to elevated CA19-9 and the presence of pancreas lesion on May 6, 2019. She was pathologically diagnosed as pT2N0 pancreatic cancer. Lung metastasis was found after 5 months during routine post-surgery follow-up. S-1 monotherapy was given as the first-line chemotherapy starting from December 6, 2019. During the surgical area palliative radiation, tumor progression was observed in the lung.

Since the CLDN18.2 IHC of 3+, 60% (Additional file 1: Figure S1), the patient was enrolled in CT041 clinical trial. Bridging chemotherapy was not given due to low tumor burden. Lymphodepletion regimen was fludarabine, cyclophosphamide and nab‐paclitaxel. A CT041 dose of 250 × 10^6^ cells was administered to the patient on July 12, 2021 (Additional file 2: Figure S2). Patient experienced grade 2 CRS (Additional file 3: Figure S3), which was further controlled with tocilizumab. PR was reached since the first evaluation 4 weeks after infusion. Target lesions of lung metastasis subsequently disappeared and achieved complete response (Fig. 1B). The tumor was still well controlled until the last follow-up on July 18, 2023.

A rapid increase in the CAR copy number was observed on D1 and further reached the peak value on D14, which was further maintained up to week 12. An increase of Treg cells and CD8+ T cells and a decrease in CD4+ T cells were observed 1 month after CT041 infusion. Both B cells and Treg cells started to recover since week 3 (Additional file 5: Table S1). Elevating IL-8 and declining TGF-β1 were both consistent with case 1, which were contradictory to the patterns of IL-6 and ferritin levels (Fig. 2).

## Discussion

Pancreatic cancer has a poor prognosis and represents urgent need for efficacious treatments. The efficacy of chemotherapy is extremely limited. After the failure of chemotherapy, both patients underwent CLDN18.2 CART therapy. PR was reached in both cases, which astonished us.

Different response patterns of lung metastasis and primary site to CT041 were observed, which may be due to the high dense stroma of the primary site and high feasibility for physical contact of the lung metastasis [4–6]. Increasing IL-6 and ferritin level was in line with the clinical manifestation of CRS and was contrary to TGF-β1, which indicated the immune regulation. Compared with persistence of cytokine, peripheral immune alteration was more lasting, involving the phenotype changes for multiple immune cells. Further studies are still needed to figure out the immune process and detailed biomarker.

### Supplementary Information

**Additional file 1. Figure S1**. Immunohistochemistry analysis of CLDN18.2 using different microscopic magnifications 10× (left) and 40× (right) for case 1 and case 2.

**Additional file 2. Figure S2**. The FACS results of CT-041 products identifying the cell subtypes of Case 1 (A) and Case 2 (C). B and D represent the phenotypes of CAR-CLDN18.2 positive cells in the products.

**Additional file 3. Figure S3**. The body temperature (A), CRP change (B) and clinical response summary (C) of the two patients.

**Additional file 4. Figure S4**. The diagram of patient enrollment and management for CT-041 administration.

**Additional file 5. Figure S5**. Table S1: markers for peripheral lymphocytes FACS

**Additional file 6.** Methods.

## Data Availability

Data of the two patients presented here were applicable.
