# Supplementary material for: CT041 CAR T cell therapy for Claudin18.2-positive metastatic pancreatic cancer
Source: J Hematol Oncol. 2023 Sep 9;16:102. doi: 10.1186/s13045-023-01491-9 (PMC10492318; doi:10.1186/s13045-023-01491-9)
Supplement: Supplementary file 1 — Additional file 1. Figure S1. Immunohistochemistry analysis of CLDN18.2 using different microscopic magnifications 10× (left) and 40× (right) for case 1 and case 2. [file 13045_2023_1491_MOESM1_ESM.docx]

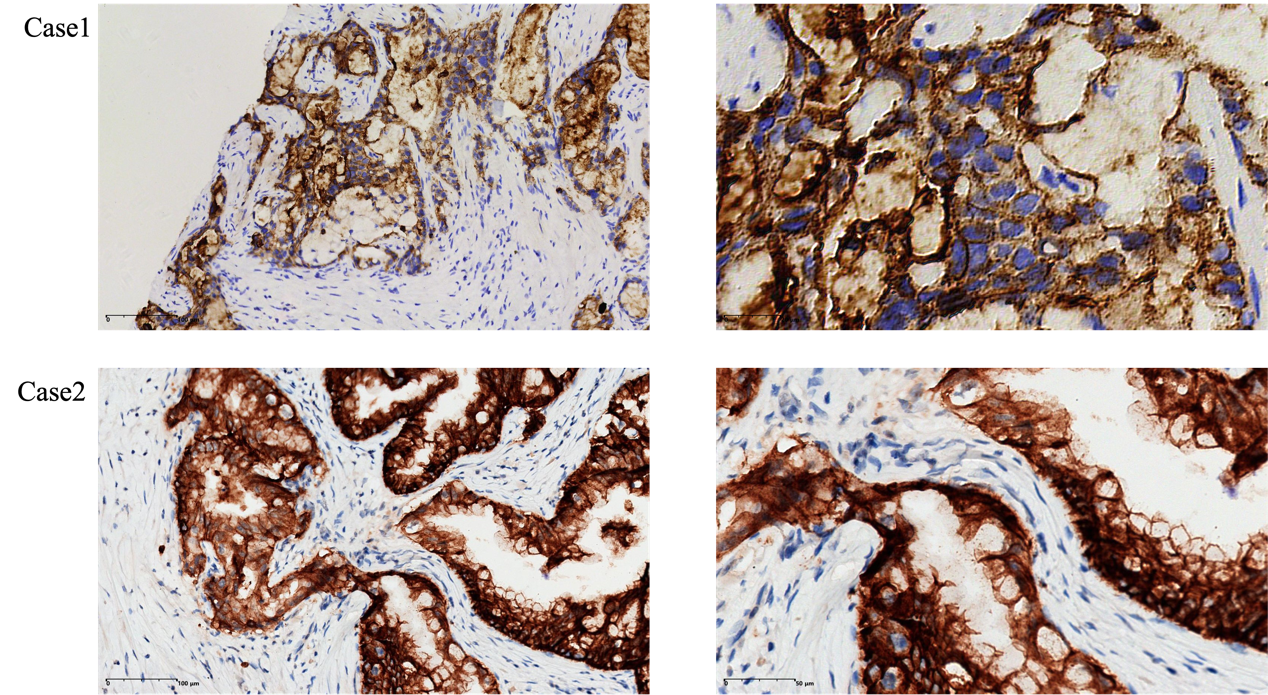


Figure S1. Immunohistochemistry analysis of CLDN18.2 using different microscopic magnifications 10× (left) and 40× (right) for case 1 and case 2.
