# Supplementary material for: CT041 CAR T cell therapy for Claudin18.2-positive metastatic pancreatic cancer
Source: J Hematol Oncol. 2023 Sep 9;16:102. doi: 10.1186/s13045-023-01491-9 (PMC10492318; doi:10.1186/s13045-023-01491-9)
Supplement: Supplementary file 2 — Additional file 2. Figure S2. The FACS results of CT-041 products identifying the cell subtypes of Case 1 (A) and Case 2 (C). B and D represent the phenotypes of CAR-CLDN18.2 positive cells in the products. [file 13045_2023_1491_MOESM2_ESM.docx]

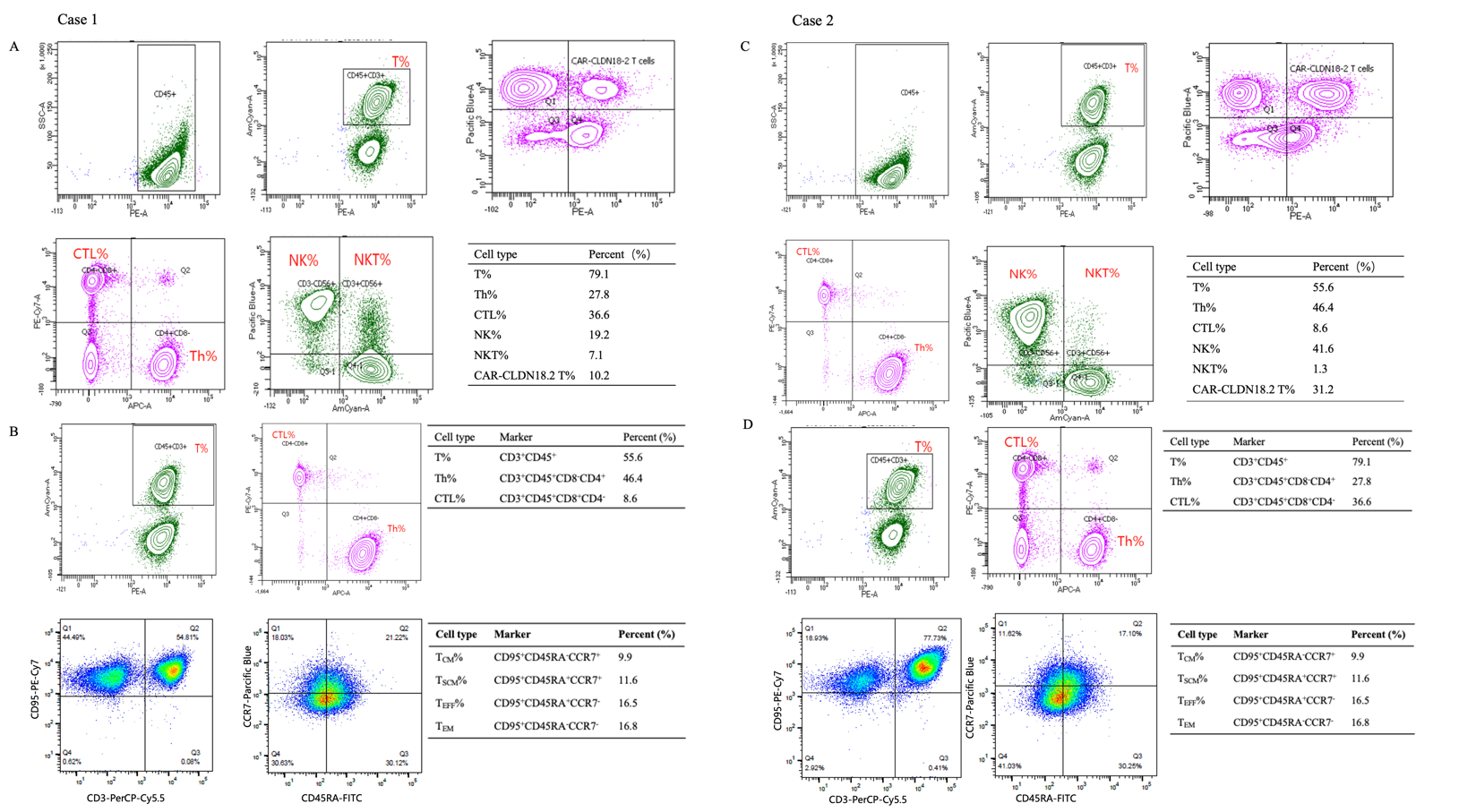


Figure S2. The FACS results of CT-041 products identifying the cell subtypes of Case 1 (A) and Case 2 (C). B and D represent the phenotypes of CAR-CLDN18.2 positive cells in the products.
