# Supplementary figures and images for: CT041 CAR T cell therapy for Claudin18.2-positive metastatic pancreatic cancer
Source: J Hematol Oncol. 2023 Sep 9;16:102. doi: 10.1186/s13045-023-01491-9 (PMC10492318; doi:10.1186/s13045-023-01491-9)

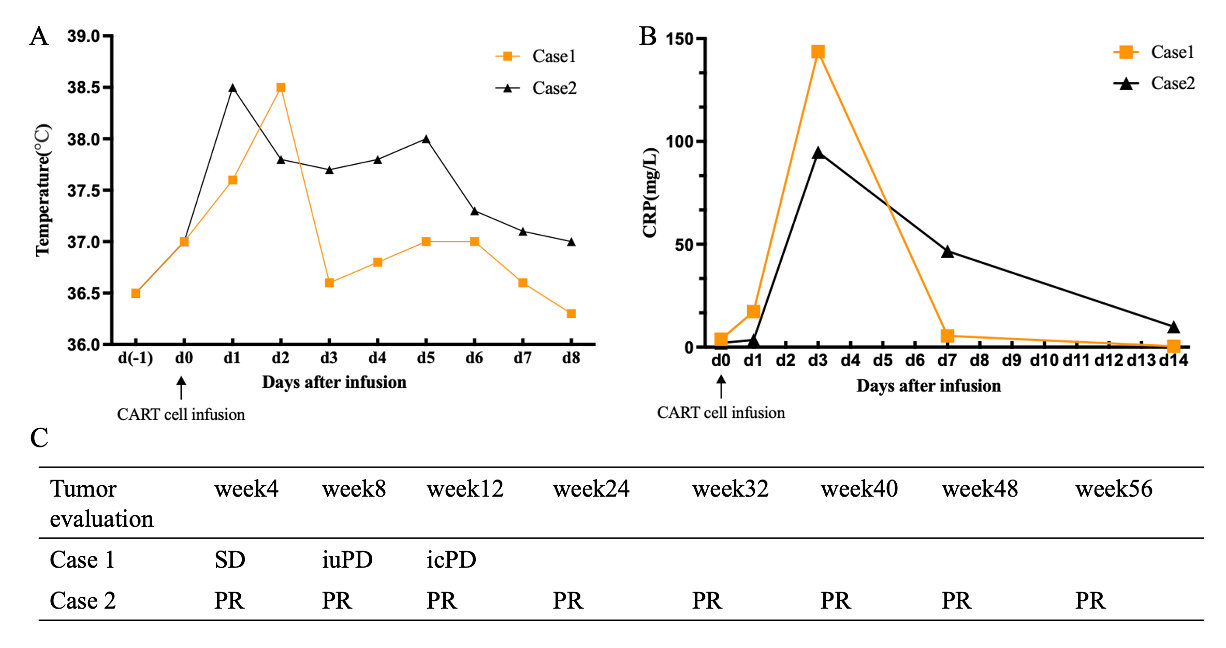
Figure S3. The body temperature(A), CRP change(B) and clinical response summary(C) of the two patients.

Supplement: Supplementary file 3 — Additional file 3. Figure S3. The body temperature (A), CRP change (B) and clinical response summary (C) of the two patients. [file 13045_2023_1491_MOESM3_ESM.docx]

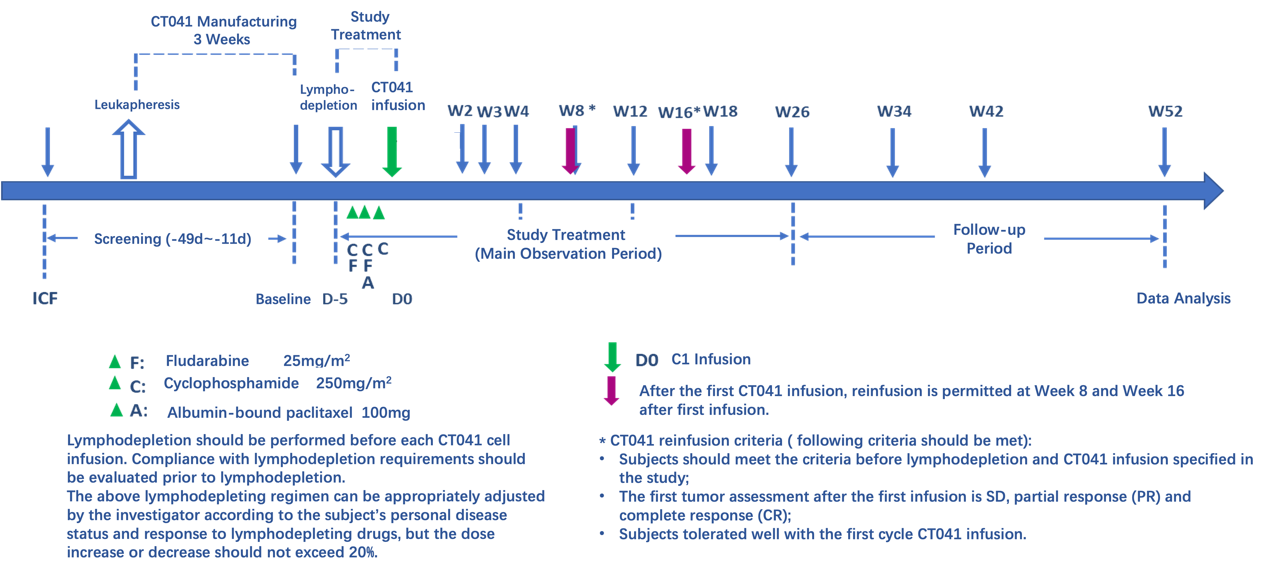


Figure S4. The diagram of patient enrollment and management for CT-041 administration.

Supplement: Supplementary file 4 — Additional file 4. Figure S4. The diagram of patient enrollment and management for CT-041 administration. [file 13045_2023_1491_MOESM4_ESM.docx]
