# Supplementary material for: CT041 CAR T cell therapy for Claudin18.2-positive metastatic pancreatic cancer
Source: J Hematol Oncol. 2023 Sep 9;16:102. doi: 10.1186/s13045-023-01491-9 (PMC10492318; doi:10.1186/s13045-023-01491-9)
Supplement: Supplementary file 5 — Additional file 5. Figure S5. Table S1: markers for peripheral lymphocytes FACS [file 13045_2023_1491_MOESM5_ESM.docx]

Table S1: markers for peripheral lymphocytes FACS

| Markers | Clone |
| --- | --- |
| CD3 | SK7 |
| CD4 | SK3 |
| CD8 | SK1 |
| CD19 | SJ25C1 |
| CD25 | 2A3 |
| CD28 | L293 |
| CD127 | HIL-7R-M21 |
