# Supplementary material for: CT041 CAR T cell therapy for Claudin18.2-positive metastatic pancreatic cancer
Source: J Hematol Oncol. 2023 Sep 9;16:102. doi: 10.1186/s13045-023-01491-9 (PMC10492318; doi:10.1186/s13045-023-01491-9)
Supplement: Supplementary file 6 — Additional file 6. Methods. [file 13045_2023_1491_MOESM6_ESM.docx]

**Methods**

**Generation of CT041**

After apheresis, peripheral blood mononuclear cell (PBMCs) from patients were collected and further purified by Ficoll density gradient centrifugation. Then, human IL-2 was added into PBMCs culture medium. After CD3/CD28 Dynabeads activation, T cells were transduced with lentiviral vector encoding the *CAR-CLDN18.2*. CAR T cells were harvested and formulated when cell quantity reached the dose requirement. fluorescence-activated cell sorting (FACS) was performed before product release (Additional file 2: Figure S2).

**qPCR analysis of CT041 expansion and persistence**

The persistence of CAR T cells in peripheral blood was determined by quantification of the woodchuck hepatitis post-transcriptional regulatory element region of the lentiviral transgene by qPCR. Genomic DNA (gDNA) was extracted using the QIAamp DNA Midi Kit (Qiagen). The standard curve for the transcript copy number was established by the amplification of a ten-fold serially diluted linearized plasmid PSD001 between 2 × 10^6^ and 200 copies. The number of transgene copies per microgram of gDNA was determined on a 7500 Fast (Thermo Fisher Scientific) triplicated for each sample.

**Peripheral blood biomarker testing**

Flow cytometry (BD FACSCantoII) were then performed to identify dynamic alteration of peripheral lymphocytes subclones every ~30 days, basing on classic immune cell markers (Additional file 5: Table S1). Dynamic cytokines were examined on D0, D3, D7, D10, D14, W3, W4, and every 4 weeks thereafter. IL-6, IL-8, IL-10 and IFN-γ were examined based on FACS CBA assay (BD FACSCanto Plus). IL-2 and IL-15 were examined by electrochemiluminescence MSD assay (MSD QuickPlex SQ120). ELISA was used for TGF-β test (Biotek Synergy LX).
